# Case Report: Serum sickness induced by dupilumab—clinical insights and Olinkproteomic analysis

**DOI:** 10.3389/fimmu.2025.1512482

**Published:** 2025-06-10

**Authors:** Hongguo Zhu, Qian’e Huang, Linhong Zheng, Wencui Yao, Jumei Xia

**Affiliations:** Department of Nephrology, The Fourth Affiliated Hospital of Guangzhou Medical University, Guangzhou, China

**Keywords:** dupilumab, serum sickness, inflammatory cytokines, proteomics, immunological mechanism

## Abstract

Dupilumab is a humanized monoclonal antibody that targets interleukin-4 (IL-4) and IL-13 pathways, commonly used in the treatment of various inflammatory conditions. Despite its therapeutic efficacy, it can also lead to rare adverse reactions, including serum sickness. Studies exploring serum sickness related to dupilumab is sparse. This report presents a case involving a middle-aged woman who developed a series of symptoms after receiving dupilumab for dermatitis, which ultimately resulted in a diagnosis of serum sickness. In this case, the patient with serum sickness exhibited a range of symptoms, including fever, rash, arthralgia, and myalgia, as well as thrombotic events, transient microscopic hematuria, and abnormal signals in the spleen and kidneys. A proteomic analysis of 92 inflammatory factors in the patient’s serum was performed using Olink technology, revealing significant elevations in several cytokines, including IL-8, IL-6, Caspase-8 (CASP-8), IL-22 receptor subunit alpha-1 (IL-22 RA1), IL-5, IL-24, IL-17A, IL-20 receptor subunit alpha (IL-20RA), IL-2 receptor subunit beta (IL-2RB), and IL-10 receptor subunit alpha (IL-10RA) during the course of serum sickness. This report contributes to the existing knowledge by detailing the clinical manifestations and cytokine profile of serum sickness induced by dupilumab and suggests potential immunological mechanisms underlying this rare adverse reaction. The findings underscore the need for awareness of such reactions and provide a foundation for future research into targeted interventions.

## Introduction

Serum sickness, a type III hypersensitivity reaction mediated by immune complexes, typically occurs 5–7 days after the injection of heterologous serum or proteins (1, 2). The primary symptoms include fever, rash, polyarthritis, lymphadenopathy, and discomfort, with less common features such as headaches, edema, enlarged spleen, blurred vision, glomerulonephritis, as well as various gastrointestinal symptoms such as abdominal distension, cramps, nausea, vomiting, and peripheral neuropathy (3, 4).

Dupilumab, a humanized monoclonal antibody, selectively inhibits the inflammatory signaling pathways mediated by interleukin-4 (IL-4) and IL-13, and is widely used in clinical practice for the treatment of atopic dermatitis and asthma (5, 6). It has been confirmed by multiple clinical studies as a safe and effective treatment (7). Commonly reported adverse effects associated with dupilumab include reactions at the injection site, conjunctivitis, eosinophilia, and upper respiratory infections. However, occurrences of serum sickness have been exceptionally rare, with only two cases documented to date (8, 9).

In this report, we present a unique case of serum sickness following the treatment of atopic dermatitis with dupilumab. This case was further investigated through a proteomic analysis employing a 92-plex inflammation panel, which elucidated significant alterations in the inflammatory cytokine profiles. This analysis provides deeper insight into the immunological mechanisms possibly triggered by dupilumab in the context of serum sickness, thereby enriching our understanding and management of rare adverse reactions to biologic therapies.

## Case presentation

**Figure 1:**
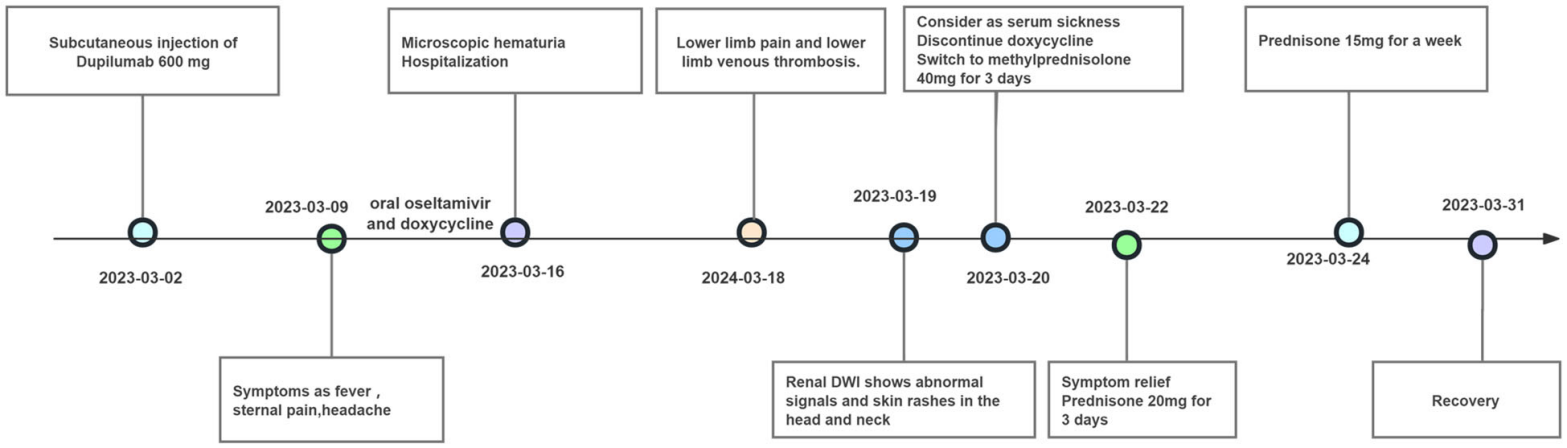
The timeline of symptom onset and treatment for the patient.

A 49-year-old female patient with a history of atopic dermatitis, who had not received any systemic or topical treatments for approximately ten months, received her first subcutaneous injection of dupilumab (600 mg) on March 2, 2023, as part of standard therapy for moderate-to-severe atopic dermatitis. One week after the first injection, she developed persistent fever accompanied by transient sore throat, sternal pain, headache, and recurrent erythema in a stripe-like or flaky pattern on both thighs. The erythema could spontaneously resolve, but she also experienced lumbosacral pain. Despite treatment with oral oseltamivir and doxycycline, along with antipyretic treatment using celecoxib, symptoms such as fever, headache, and lumbosacral pain persisted. Subsequently, a series of diagnostic examinations were conducted. Laboratory evaluations revealed normal leukocyte and neutrophil counts, along with a normal erythrocyte sedimentation rate. C-reactive protein level was mildly elevated, ranging from 17.6 to 25.3 mg/L (normal range 0–5 mg/L), and lactate dehydrogenase level was increased, recorded between 321 and 464 IU/L (normal range 80–285 IU/L). Urinalysis indicated transient microscopic hematuria in the absence of proteinuria. The complete progression of the disease and remission in this patient is illustrated in **Figure 1**.

Investigations for infectious etiology yielded negative results across multiple tests: blood and urine cultures, blood metagenomic next-generation sequencing (mNGS), tuberculosis interferon release assay, nucleic acid tests for novel coronavirus, influenza A/B throat swabs, dengue antigen, epidemic hemorrhagic fever antibody, cytomegalovirus IgM antibody, and tests for rickettsia and Toxoplasma gondii DNA.

**Figure 2:**
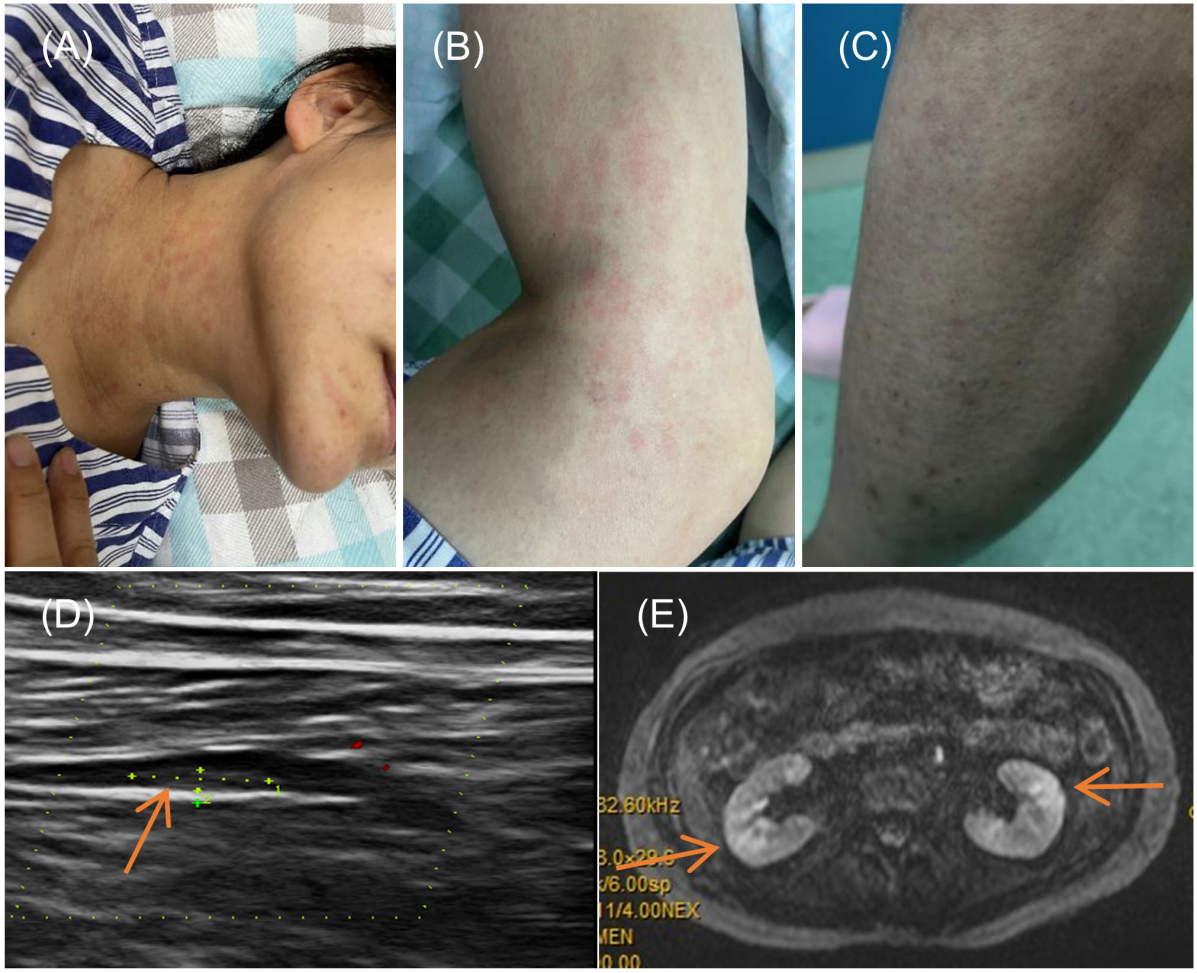
Symptoms and Imaging Results of the Patient. **(A, B)** Rash on the neck and arms of the patient; **(C)** Change in skin color of the lower limbs upon standing; **(D)** Doppler ultrasound of the lower limbs indicating local thrombus formation; **(E)** Diffusion-weighted imaging (DWI) sequence from an enhanced MRI of the kidneys.

**Figure 3:**
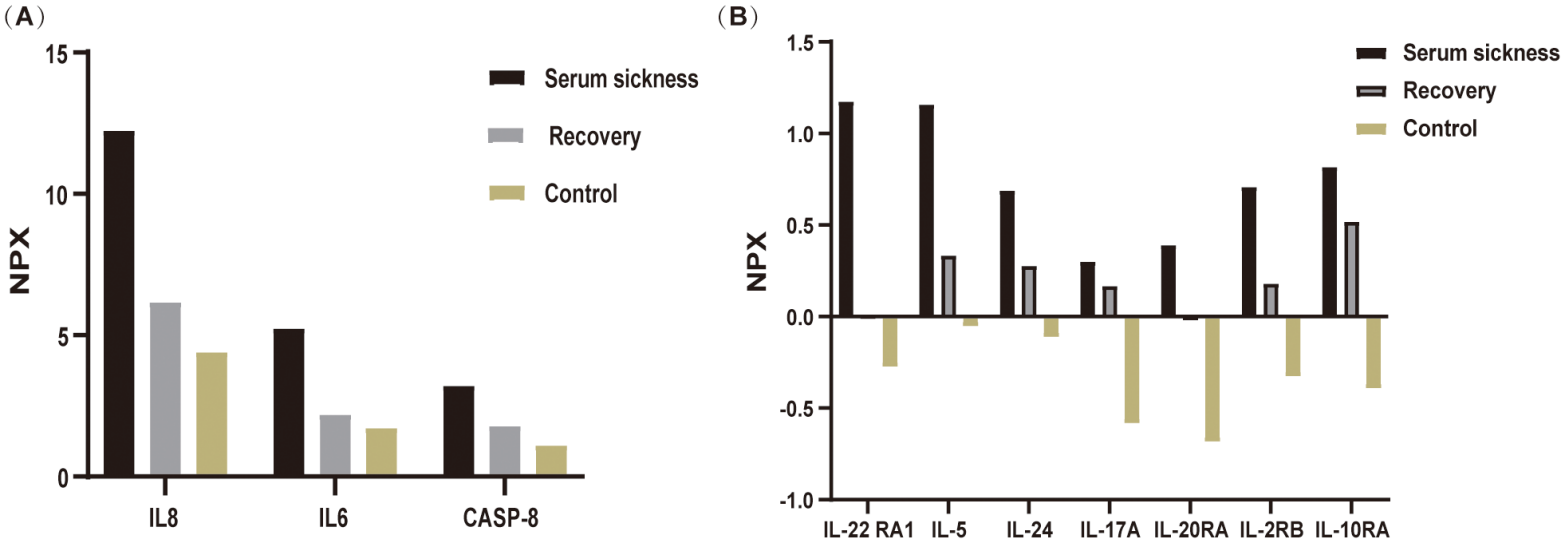
Differential expression of inflammatory cytokines during disease progression analyzed using Olink proteomics. Blood samples were collected from patient and analyzed via Olink’s multiplex proteomics, targeting 96 inflammatory markers. **(A)** illustrates the variations in inflammatory cytokines IL-8, IL-6, and CASP-8 across three groups: the Serum Sickness phase, the Recovery phase, and the Healthy Control group (mean of three individuals. **(B)** depicts the changes in the inflammatory factors IL-22RA1, IL-5, IL-24, IL-17A, IL-20RA, IL-2RB, and IL-10RA among these three groups. Samples from the serum sickness group were obtained at the peak of the disease, samples from the Recovery group were collected one-month post-disease, and Control group samples were taken from three healthy volunteers.

In the realm of rheumatology and immunology, antinuclear antibodies, extractable nuclear antigen (ENA) antibody profile, and complement levels were found to be within normal limits. Evaluations of metabolic parameters and thyroid function, as well as assessments for female tumor markers, showed no abnormalities. Imaging studies, including chest and abdominal CT scans, did not reveal any etiological findings. Whole-body PET-CT imaging revealed multiple slightly enlarged lymph nodes in the cervical, right supraclavicular, mediastinal, left axillary, and bilateral inguinal regions, all showing increased metabolic activity suggestive of inflammatory reactive changes. Additional findings included splenomegaly and multiple hypermetabolic foci in the kidneys, raising the possibility of systemic immune-related disorders. Enhanced MR imaging of the kidneys also demonstrated abnormal signals in the diffusion-weighted imaging (DWI) sequences (**Figure 2E**). The DWI sequences showed slightly high signal intensity, while the apparent diffusion coefficient (ADC) map displayed slightly low signal intensity, with ADC values ranging from 1.866 to 1.930 × 10⁻^3^ mm²/s. Bone marrow biopsy excluded hematologic malignancies. As the patient’s condition progressed, pain developed in both lower extremities within 1–2 minutes of standing, severely limiting the ability to walk a few meters. With prolonged standing, darkening of the skin in the lower extremities was observed (**Figure 2C**), and Doppler ultrasound demonstrated a slowdown in blood flow with the formation of local thrombi (**Figure 2D**). Notably, plasma D-dimer levels were elevated from 0.6 to 2.09 mg/L, exceeding the normal range of 0-0.5 mg/L. Concurrently, rashes developed on the patient’s head, neck, and arms, as depicted in **Figures 2A, B**. After excluding other conditions, the patient was diagnosed with Dupilumab-related serum sickness. Blood samples were collected on March 19, 2023, which was the third day of hospitalization and approximately two weeks after symptom onset, for analysis of 92 inflammatory proteins. The analysis revealed that during the serum disease phase, there was a significant elevation in the levels of certain inflammatory cytokines, including IL-8, IL-6, Caspase-8 (CASP-8),IL -22 receptor subunit alpha-1 (IL-22 RA1), IL-5, IL-24, IL-17A, IL-20 receptor subunit alpha (IL-20RA), IL-2 receptor subunit beta (IL-2RB), and IL-10 receptor subunit alpha (IL-10RA) (**Figure 3**).

Through daily intravenous administration of 40 mg methylprednisolone and subcutaneous injections of enoxaparin, symptoms such as headaches, sacral pain, lower limb pain, and rashes were effectively alleviated in the patient. The dosage of methylprednisolone was progressively reduced after three days and continued over a period of two weeks, leading to the patient’s full recovery.

## Discussion

The advent of biological therapies has transformed the management of various autoimmune and inflammatory diseases, offering targeted intervention with generally favorable safety profiles. Dupilumab has shown remarkable efficacy in the treatment of various allergic and inflammatory conditions, including atopic dermatitis and asthma. However, the emergence of rare but potentially serious adverse events such as serum sickness underscores the importance of understanding the immunological mechanisms underlying these reactions. In this case, we integrate clinical observations and proteomic profiling data to elucidate the pathophysiology of dupilumab-induced serum sickness and discuss its implications for clinical management and future research.

Our patient exhibited fever, headache, substernal pain, myalgias, rash, and transient microscopic hematuria following dupilumab therapy aligns with previous reports of serum sickness associated with other biologic agents (10–12). The clinical presentations observed included splenomegaly, renal abnormalities, decreased blood flow in the lower extremities, and a hypercoagulable state, which collectively suggest a systemic inflammatory response triggered by dupilumab. To further assess the causality of the adverse reaction in this patient, we applied the Naranjo Adverse Drug Reaction Probability Scale. Based on this scale, the total score was 6, indicating a possible causal relationship between Dupilumab and the serum sickness observed in this patient. This supports the hypothesis that Dupilumab may have contributed to the onset of serum sickness.

Prior literature includes two documented instances of serum sickness following dupilumab administration. One case presented solely with fever (9), while another exhibited more complex symptoms, including muscle pain, facial and hand swelling, joint pain, and a rash at the injection site with elevated inflammatory markers, and was diagnosed with serum sickness one day after the second dose (8). As of April 2024, the OpenVigil database (13) recorded 62 cases of serum sickness associated with dupilumab, predominantly in middle-aged females with either dermatitis or asthma. Consistent with our clinical observation, reported symptoms encompassed rash, arthralgia, fever, headache, and peripheral edema, emphasizing the need for heightened vigilance in monitoring patients receiving dupilumab therapy. This case study was comprehensive, revealing several clinical manifestations that are less commonly observed in other instances of serum sickness. These included splenomegaly, abnormal renal imaging signals (bilateral diffusion-weighted imaging hyperintensity with apparent diffusion coefficient values of 1.866-1.930×10⁻³ mm²/s, suggesting acute inflammatory parenchymal changes (14)), and a hypercoagulable state. These symptoms not only expand the spectrum of clinical manifestations of serum sickness but also indicate a systemic inflammatory response triggered by dupilumab.

Proteomic profiling using Olink technology identified a distinct panel of inflammatory cytokines significantly elevated during serum sickness, including IL-8, IL-6, CASP-8, IL-22 RA1, IL-5, IL-24, IL-17A, IL-20RA, IL-2RB, and IL-10RA. The dysregulation of these cytokines suggests a complex interplay between Th1/Th2 immune responses and proinflammatory cytokine cascades in dupilumab-induced serum sickness. Specifically, IL8 and IL6 are known pro-inflammatory markers associated with acute inflammation and have been frequently observed in various autoimmune and inflammatory conditions (15–18). Their marked increase in patient correlates with the severity of symptoms like fever and pain, aligning with findings from Wang et al., who noted that the upregulation of IL-6, IL-8 expression contributes to the development of acute inflammation and inflammatory pain (15). CASP-8, a crucial mediator of apoptosis and inflammation, was notably elevated. Although CASP-8 has been extensively studied in conditions involving apoptosis (19) and inflammatory responses, such as sepsis and autoimmune diseases (20), its role in serum sickness has not been well documented. The elevation of CASP-8 in this patient suggests a potential novel mechanism contributing to the pathophysiology of serum sickness, which is characterized by immune complex-mediated inflammation. This finding is important as it provides new insights into the molecular pathways involved in serum sickness, and may help identify potential biomarkers for disease severity as well as new therapeutic targets. An elevation in IL-10RA, a key receptor for IL-10, indicates a compensatory response of the immune system, while the concurrent increase in IL-22RA1 suggests that complex regulatory mechanisms are employed by the body to combat inflammation (21, 22). The elevation of Th2-Associated Cytokines (IL-5, IL-24) typically associated with allergic and atopic conditions could explain the allergic manifestations like skin rash and pruritus observed in the patient. Endothelial cells are activated by IL-17A, which induces the upregulation of IL-6, IL-8, and intercellular adhesion molecule-1 (ICAM-1) (23). This upregulation contributes to enhanced tissue inflammation and procoagulant activity, thereby facilitating vascular activation. In this context, the observed hypercoagulable state and the slowing of blood flow in the lower extremities can be explained by the formation of localized thrombi, which partially obstruct the vascular channels. Additionally, the elevated inflammatory cytokines, including IL-6, IL-8, and IL-17A, may further exacerbate endothelial dysfunction. This dysfunction, combined with increased procoagulant activity, impairs normal blood flow dynamics, leading to reduced circulation in the affected areas. The interplay between thrombosis and inflammation thus contributes to the observed slowing of blood flow, consistent with findings in other inflammatory conditions where cytokine-driven vascular changes have been reported (24–26).

The observed changes in inflammatory biomarkers provide valuable insights into the pathophysiology of dupilumab-induced serum sickness and have important implications for clinical management. Monitoring of serum cytokine levels may aid in early detection of serum sickness and facilitate timely intervention to mitigate disease progression. However, this approach faces certain clinical limitations, including high costs, limited accessibility of multiplex cytokine platforms, and the absence of standardized thresholds for interpretation, which currently restrict its use in routine clinical practice. Thus, cytokine profiling should be considered an adjunctive tool pending further validation. Furthermore, targeting specific cytokines implicated in serum sickness, such as IL-6 or IL-8, may represent novel therapeutic strategies to modulate immune dysregulation and alleviate symptoms in affected patients. However, further research is warranted to validate these findings and assess the efficacy and safety of targeted immunomodulatory therapies in the management of serum sickness.

Despite the insights gained from our study, several knowledge gaps remain to be addressed. Firstly, the precise mechanisms by which dupilumab triggers serum sickness require further elucidation, including the interplay between IL-4/IL-13 blockade and downstream immune responses. Additionally, the long-term implications of serum sickness on disease course and treatment outcomes need further investigation, particularly in patients receiving chronic dupilumab therapy. Finally, the utility of inflammatory biomarkers as predictive or prognostic indicators of serum sickness requires validation in larger prospective studies.

## Conclusion

In conclusion, this study provides comprehensive insights into the clinical manifestations and inflammatory biomarkers associated with dupilumab-induced serum sickness. By integrating clinical data and protein profiling, our research aims to explore the immunological mechanisms underlying this rare adverse reaction and identify potential therapeutic targets for future research. Our findings underscore the importance of vigilance in monitoring patients receiving dupilumab therapy and highlight the need for further research to optimize clinical management and improve patient outcomes.

## Data Availability

The original contributions presented in the study are included in the article/supplementary material. Further inquiries can be directed to the corresponding author.
